# Repeatable Construction Method for Engineered Zinc Finger Nuclease Based on Overlap Extension PCR and TA-Cloning

**DOI:** 10.1371/journal.pone.0059801

**Published:** 2013-03-25

**Authors:** Wataru Fujii, Kiyoshi Kano, Koji Sugiura, Kunihiko Naito

**Affiliations:** 1 Department of Animal Resource Sciences, Graduate School of Agricultural and Life Sciences, University of Tokyo, Tokyo, Japan; 2 Laboratory of Developmental Biology, Joint Faculty of Veterinary Medicine, Yamaguchi University, Yamaguchi, Japan; 3 Biomedical Science Center for Translational Research (BSCTR), United Graduate School of Veterinary Science, Yamaguchi University, Yamaguchi, Japan; Ohio State University Comprehensive Cancer Center, United States of America

## Abstract

Zinc finger nuclease (ZFN) is a useful tool for endogenous site-directed genome modification. The development of an easier, less expensive and repeatedly usable construction method for various sequences of ZFNs should contribute to the further widespread use of this technology. Here, we establish a novel construction method for ZFNs. Zinc finger (ZF) fragments were synthesized by PCR using short primers coding DNA recognition helices of the ZF domain. DNA-binding domains composed of 4 to 6 ZFs were synthesized by overlap extension PCR of these PCR products, and the DNA-binding domains were joined with a nuclease vector by TA cloning. The short primers coding unique DNA recognition helices can be used repeatedly for other ZFN constructions. By using this novel OLTA (OverLap extension PCR and TA-cloning) method, arbitrary ZFN vectors were synthesized within 3 days, from the designing to the sequencing of the vector. Four different ZFN sets synthesized by OLTA showed nuclease activities at endogenous target loci. Genetically modified mice were successfully generated using ZFN vectors constructed by OLTA. This method, which enables the construction of intended ZFNs repeatedly and inexpensively in a short period of time, should contribute to the advancement of ZFN technology.

## Introduction

Zinc finger nuclease (ZFN) is an artificial restriction enzyme that causes DNA double-strand breaks (DSB) on a specific locus of genome sequences, and is useful for genome editing by error-prone non-homologous-end-joining (NHEJ) or homologous recombination with exogenous DNA [1], [2]. ZFN is reported to function in various cells including embryos and to be able to generate vertebrates, plants and insects with specific genetic modifications [3]–[13].

ZFNs consist of C2H2 zinc finger (ZF) domains and a FokI-derived DNA endonuclease domain. Paired ZFNs bind to the plus and minus strands of the target locus with 5–6 bp gaps [14] and digest DNA by dimerized nucleases [15]. A ZF domain binds to a specific DNA triplet through its 7-amino-acid DNA-recognition helix and a different combination of 7 amino acids will bind to a different DNA triplet. By combining multiple ZF domains with different recognition helices, ZFNs are able to bind specifically to arbitrarily chosen DNA target sites [16], [17]. The relationship between the amino acid sequence of the DNA recognition helix and the target DNA triplets has been widely investigated [14], [16]–[19], and website tools for designing the appropriate ZF against endogenous DNA sequences have been developed [20]–[23] However, the lack of activity against endogenous target sites have been recently pointed out in ZFNs designed by these webstites. It is difficult to design an exclusive ZFN, which functions with selectivity at each unique target site. Therefore, it is beneficial to be able to design several ZFN pairs against several sites within one target gene. This makes developing an easy and efficient ZFN construction method very important.

At present, two major construction methods are being commonly used: one is the overlapping of synthetic long oligonucleotides by PCR [16] and the other is the assembly of ZF modules from a prepared ZF-coding plasmid DNA library through consecutive restriction and ligation reactions [24], [25]. In the former method, an approximately 300 bp DNA sequence coding 3 ZF DNA-binding domains is divided into several synthetic long (a few dozen bp) oligonucleotide fragments, which have overlap sequences with the adjacent fragments in both ends, and these fragments are combined by PCR using the overlap sequences. Carroll et al. formulated a method to construct ZFNs consisting of 3 ZFs by overlapping 4 or more synthetic long oligonucleotides (60 bp<). They reported that the design, construction and cloning could be completed within about two weeks if all steps went smoothly, and expression and testing could be completed in an additional week [16]. Osborn et al. improved this method to shorten the construction period from design to testing to 1 week by constructing a specific expression vector consisting of nucleases and bound it with the prepared ZFs by enzymatic recombination [26]. However, the number of ZFs in each array was confined to 3 (ZF1, ZF2 and ZF3) in these reports, and the overlap sequences of ZF1, ZF2 and ZF3 were selected from different sites of the ZF domain in order to combine them into the correct order at once with PCR. In other words, an oligonucleotide containing the DNA-recognition helix of ZF1 could not be used for a different position, i.e. ZF2 or ZF3, and as a result, renewed multiple long oligonucleotides were required for constructing a new ZF in a different position even if its DNA-recognition helix is the same. In another method called ZF modular assembly, the desired ZF module is cut out from the ZF-module-library vector by a restriction enzyme, and the ZF module is connected sequentially one by one. In this method, all ZF modules can be used interchangeably, however, consecutive restriction and ligation reactions are complex and time consuming. As an alternative to self-construction methods, ready-made or custom-made ZFNs can be purchased commercially, but doing so is costly and requires a long time [27]. Due to the above reasons, the previously reported ZFN-preparation methods have both merits and demerits in terms of cost, time, labour, and repeatability. Therefore, the establishment of a novel inexpensive, rapid and simple method for ZFN construction may be necessary for ZFN technology to reach its full potential.

In the present study, we attempted to establish a novel ZFN construction method. As different parts of various ZFs were confined within the DNA recognition helix, we designed short PCR primers (<30 bp) corresponding to the helix to try to synthesize each ZF by PCR, and combined 4–6 ZFs by overlap extension PCR. Furthermore, in order to connect these PCR-derived ZFs and nuclease vector rapidly by TA cloning without the use of restriction enzymes, a nuclease-coding platform vector which could be used as a TA vector for ZFN construction was designed and synthesized. For this overlap extension PCR and TA-cloning method, which we named “OLTA”, we designed and constructed 4 sets of ZFN vectors against 2 reported loci, *Rosa26* [28] and Interleukin 2 receptor, gamma chain (*Il2rg*) [29], and 2 original loci, GLI-Kruppel family member GLI3 (*Gli3*) and cyclin-dependent kinase inhibitor 1B (*Cdkn1b*), each within a single day and finished until the *E. coli* transformation process, or within three days including the colony selection and the sequencing procedure of the ZFN vectors. Moreover, the functions of the constructed ZFNs were evaluated by injecting them into the mouse embryos, and site-directed mutagenesis of endogenous target loci was examined. Then, we attempted to generate a genetically modified mouse using a set of synthesized ZFNs.

## Materials and Methods

### Ethics Statement

All animal care and experiments conformed to the Guidelines for Animal Experiments of The University of Tokyo, and were approved by the Animal Research Committee of The University of Tokyo.

### Construction of Platform Vectors

**Figure 1:**
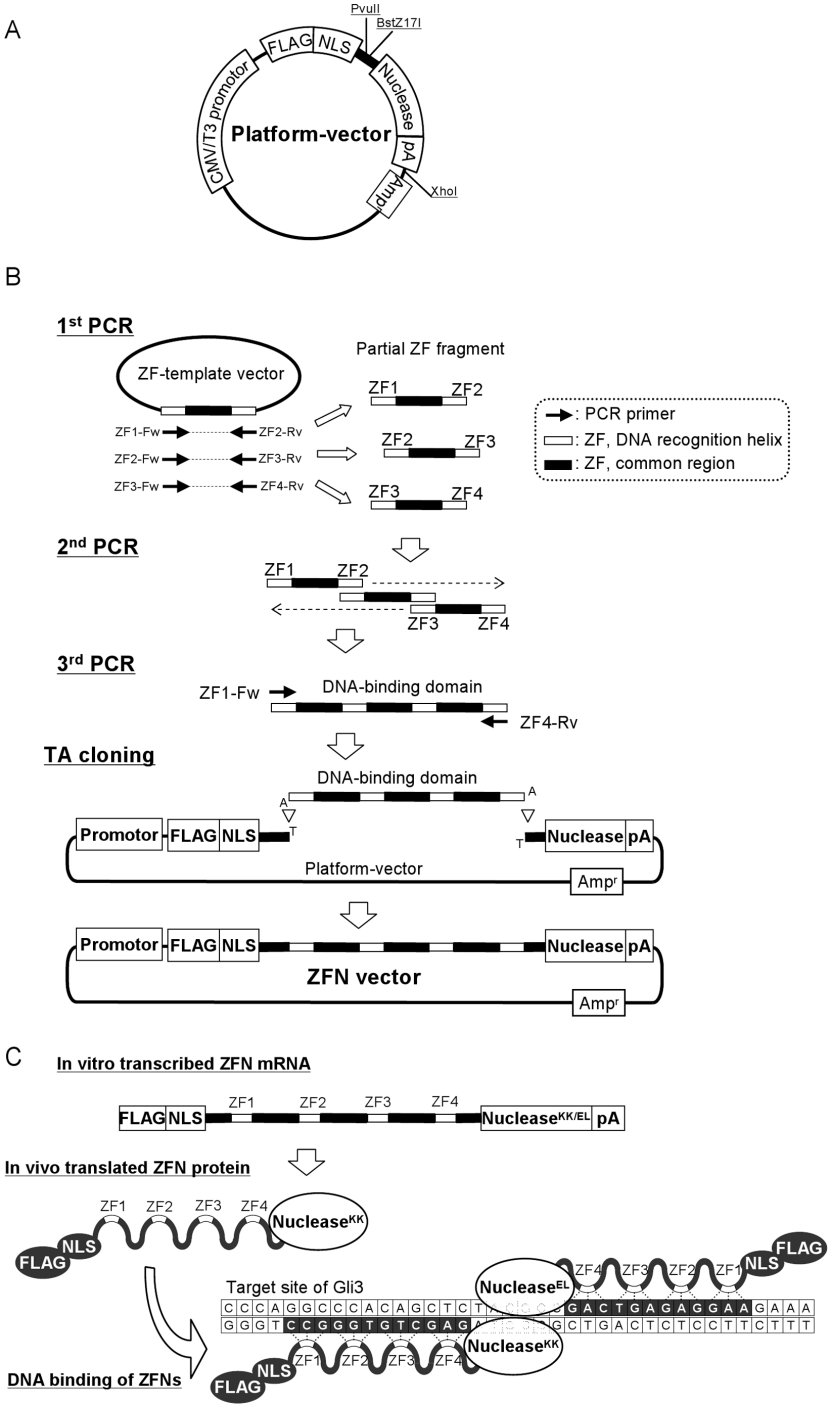
Experimental schemes for vector construction. (A) Composition of the platform vector used for ZFN construction. (B) Construction procedures for ZFN vectors by OLTA. In this figure, construction of a DNA-binding domain composed of 4 ZFs is shown as an example. Three partial ZF fragments were synthesized by the 1st PCR with the primer sets shown in Table 2 and they were combined by overlap extension PCR (2nd PCR) and amplified by 3rd PCR; then the DNA-binding domain was joined with the platform vector by TA cloning. (C) A scheme showing constructs of *in vitro* transcribed ZFN mRNA and *in vivo* translated ZFN protein, and ZFN binding with target DNA; *Gli3* target site is shown in this figure for example.

The platform-vector construct is shown in Figure 1A. A ZFN cassette consisting of the SV40 nuclear-localization sequence, the flanking sequence of the C2H2 zinc finger containing restriction sites of PvuII and BstZ17I, and the FokI-derived nuclease domain including KK or EL obligate heterodimer mutations [15] and the Sharkey mutation [30] were constructed from 12 synthetic oligonucleotides by overlap extension PCR using a thermal cycler. The ZFN cassette was digested by BglII and KpnI and was inserted into the BglII-KpnI site of a pCMV-Tag1 vector. Next, the 3′ UTR of mouse TATA box binding protein-like 1 with a 95-bp polyadenine tail, which was cloned from adult mouse testis cDNA according to previous report [31], was inserted into the KpnI site of the above vector in order to induce constitutive high expression. The vector was digested with DraII and AflIII to eliminate unnecessary restriction sites, and joined with a DraII-AflIII-digested pUC19 fragment coding the ampicillin resistance gene. Then, the constructed vector was sequenced using a commercial sequencing kit (Applied Biosystems, Foster City, CA) and a DNA sequencer (Applied Biosystems) according to the manufacturer’s instructions, and is referred to hereafter as the “platform vector”. The sequence of the platform vector is shown in Figure S1. Before TA cloning, the platform vector was digested with PvuII and BstZ17I, and ddTTP was added to the 3′ end of digested sites using terminal transferase. The treated platform vector was purified by agarose gel electrophoresis, extracted using a gel extraction kit, and stored at −20°C until use.

### Construction of ZF-template Vector

The zinc finger domain of mouse early-growth-response protein 1 (*Egr1*) has been well studied of its function and configuration [32]–[34], and has been used for ZFN in previous reports [16], [26]. In the present study, the partial sequence (104 bp) from the first to the second DNA recognition helices of *Egr1* ZF domain (1327–1431 of NM_007913) was cloned from adult mouse testis (cDNA by RT-PCR) using the appropriate forward primer (5′-CGCTCGGATGCGCTTACCCGCCATATCCG-3′) and reverse primer (5′-CGGGCAAGATTATCCGAGCGACTGAAG-3′). The PCR product was cloned into a pGEM-T Easy vector, and is referred to here as the “ZF template vector”. The ZF template vector was sequenced as described above, and the sequence of the inserted ZF domain is shown in Figure S2.

### Construction of ZFN Vector by Overlap Extension PCR and TA-cloning (OLTA)

Target sequence and DNA-recognition helices of ZFN against *Rosa26* were the same as those reported previously [28], and those against *Il2rg* were drawn from the report in rat [29] with a change of ZF2 DNA-recognition helices in the left-ZFN from TAG to GCG according to the mouse sequence (GCA GCG TGA GGT TGG). Target sites of *Gli3* and *Cdkn1b* were determined by searching the GNN triplet or reported high quality triplet [16] from their sequences. These target sequences were shown in Table 1, and an outline of ZFN vector construction is shown in Figure 1B. All PCR were performed using the primer sets shown in Table 2 on the condition of Table 3. The 3rd PCR products were cloned into the PvuII/BstZ17I digested and 3′-T-added platform vectors by TA-cloning. The left-side ZFs and the right-side ZFs were cloned into the KK platform vector and the EL platform vector, respectively, referred to hereafter as the left-ZFN vector and right-ZFN vector, respectively. After transformation of competent cells with these vectors and plating on LB plates, the colonies were subjected to colony PCR using T3-promoter primer as a forward primer and the reverse primer of 3rd PCR to confirm the correct insertion and direction of PCR products. The vectors that have the correct insertion size with true direction of PCR product were sequenced as described above.

**Table 1:** ZFN target sequences and ZF alignment of each ZFN.

Gene	Target Sequence		ZF1	ZF2	ZF3	ZF4	ZF5	ZF6
*Gli3*	Left	GAG CTG TGG GCC	GCC	TGG	CTG	GAG		
	Right	GAC TGA GAG GAA	GAA	GAG	TGA	GAC		
								
*Rosa26*	Left	AGA AAG ACT GGA GTT GCA	GCA	GTT	GGA	ACT	AAG	AGA
	Right	TGG GCG GGA GTC	GTC	GGA	GCG	TGG		
								
*Il2rg*	Left	GCA GCG TGA GGT TGG	TGG	GGT	TGA	GCG	GCA	
	Right	GGT ATG AGA AGG GGG	GGG	AGG	AGA	ATG	GGT	
								
*Cdkn1b*	Left	GCG GGT GTG GAC	GAC	GTG	GGT	GCG		
	Right	GAG GAA GAT GTC	GTC	GAT	GAA	GAG		

**Table 2:** Primer sets for 1st PCR.

Target DNA triplet	DNA-recognition-helix sequence	Forward Primer* (NDN)**		Reverse Primer* (NDN)**	
GAA	QSGNLAR	5′-	CaaTCGGgTaacCTTgCCCGCCATATCCG (7)	5′-	GGCGGGCAAGgTTAcCCGAttGACTGAAG (4)
GAC	DRSNLTR	5′-	gaCagGagTaatCTTACCCGCCATATCCG (9)	5′-	GGCGGGtAAGATTActCcTGtcACTGAAG (6)
GAG	RSDNLAR	5′-	CGCTCGGATaatCTTgCCCGCCATATCCG (4)	5′-	GGCGGGCAAGATTATCCGAGCGACTGAAG (0)
GAT	TSGNLVR	5′-	acCTCGGgTaatCTTgtCCGCCATATCCG (8)	5′-	GGCGGaCAAGATTAcCCGAGgaACTGAAG (4)
GCA	QSGDLTR	5′-	CaaTCGGgTGatCTTACCCGCCATATCCG (5)	5′-	GGCGGGtAAGATcAcCCGAttGACTGAAG (4)
GCC	DCRDLAR	5′-	gaCTGcagaGacCTTgCCCGCCATATCCG (9)	5′-	GGCGGGCAAGgTctctgcAGtaACTGAAG (9)
GCG	RSDDLQR	5′-	CGCTCGGATGacCTTcaaCGCCATATCCG (5)	5′-	GGCGttgAAGgTcATCCGAGCGACTGAAG (5)
GGA	QSGHLQR	5′-	CagTCGGgTcatCTTcaaCGCCATATCCG (9)	5′-	GGCGttgAAGATgAcCCGActGACTGAAG (7)
GGG	RSDHLSR	5′-	CGCTCGGATcacCTTtCCCGCCATATCCG (4)	5′-	GGCGGGaAAGgTgATCCGAGCGACTGAAG (3)
GGT	TSGHLVR	5′-	acCTCGGgTcacCTTgtCCGCCATATCCG (8)	5′-	GGCGGaCAAGgTgAcCCGAGgtACTGAAG (6)
GTC	DPGALVR	5′-	gaCcCGGgTGCGCTTgtCCGCCATATCCG (6)	5′-	GGCGGaCAAGcgcAtCCGgGtcACTGAAG (8)
GTG	RSDALTR	5′-	CGCTCGGATGCGCTTACCCGCCATATCCG (0)	5′-	GGCGGGtAAGcgcATCCGAGCGACTGAAG (4)
GTT	TSGSLVR	5′-	acCTCGGgTtCGtTagtCaGaCATATCCG (10)	5′-	GtCtGaCtAacgaATCCGAGgtACTGAAG (10)
AAG	RKDNLKN	5′-	CGCaaGGATaacCTTAaaaaCCATATCCG (9)	5′-	GGtttttAAGgTTATCCttGCGACTGAAG (8)
ACT	THLDLIR	5′-	acCcacctTGatCTTAtCCGCCATATCCG (10)	5′-	GGCGGatAAGATcAaggtgGgtACTGAAG (10)
AGA	QLAHLRA	5′-	CaaTtGGcTcacCTTcgCgcCCATATCCG (11)	5′-	GGgcGcgAAGgTgAgCCaAttGACTGAAG (10)
AGG	RSDHLTN	5′-	CGCTCGGATcacCTTACCaaCCATATCCG (5)	5′-	GGttGGtAAGgTgATCCGAGCGACTGAAG (5)
ATG	RRDELNV	5′-	CGCcgGGATGaGCTTAACgtCCATATCCG (5)	5′-	GGacGttAAGcTcATCCcgGCGACTGAAG (8)
CTG	RNDALTE	5′-	CGCaatGAcGCGCTTACCgaaCATATCCG (7)	5′-	GttcGGtAAGcgcgTCattGCGACTGAAG (11)
TGA	QAGHLAS	5′-	CaagCGGgTcatCTTgCCaGCCATATCCG (9)	5′-	GGCtGGCAAGATgAcCCGcttGACTGAAG (6)
TGG	RSDHLTT	5′-	CGCagtGATcacCTTACCacCCATATCCG (8)	5′-	GGgtGGtAAGgTgATCactGCGACTGAAG (8)

* The capital letters and small letters show same and different nucleotides compared to the zinc-finger-template vector.

** Number of different nucleotides compared to the zinc-finger-template vector.

**Table 3:** Overlap-extension PCR conditions.

PCR mixture	PCR conditions	
**1st PCR**		
5 µl of 10X Ex Taq Buffer	95°C, 2 min	
4 µl of dNTP Mixture (2.5 mM each)	95°C, 15 sec	X 40 cycles
2.5 µl of 10 µM Forward primer	54°C, 15 sec	
2.5 µl of 10 µM Reverse primer	72°C, 15 sec	
1 µl of 100 µg/ml Template vector	72°C, 5 min	
0.5 µl of 5 U/µl Ex Taq		
34.5 µl of Ultra pure water		
**2nd PCR**		
5 µl of 10X Ex Taq Buffer	95°C, 2 min	
4 µl of dNTP Mixture (2.5 mM each)	94°C, 30 sec	X 15 cycles
0.5 µl of 5 U/µl Ex Taq	68°C, 30 sec	
1st PCR amplicons (10 µg/ml each)	68°C, 5 min	
0.5 µl each of ZF1-ZF2 and ZF3-ZF4 amplicons and 1.5 µl of ZF2-3 amplicon (4ZF)		
or 0.5 µl each of ZF1-ZF2 and ZF4-ZF5 amplicons and 1.5 µl each of ZF2-3 and ZF3-4 amplicons (5ZF)		
or 0.5 µl each of ZF1-ZF2, ZF2-ZF3, ZF4-ZF5 and ZF5-ZF6 amplicons and 1.5 µl of ZF3-4 amplicon (6ZF)		
Adjust to 50 µl with Ultra pure water		
**3rd PCR**		
5 µl of 10X Ex Taq Buffer	95°C, 2 min	
4 µl of dNTP Mixture (2.5 mM each)	94°C, 30 sec	X 25 cycles
2.5 µl of 10 µM ZF1 Forward primer	68°C, 30 sec	
2.5 µl of 10 µM ZF4 (4ZF), ZF5 (5ZF) or ZF6 (6ZF) Reverse primer	68°C, 5 min	
0.5–1 µl of 2nd PCR reacted mixture		
0.5 µl of 5 U/µl Ex Taq		
Adjust to 50 µl with Ultra pure water		

### *In vitro* Synthesis of mRNA

For the *in vitro* synthesis of mRNAs, constructed left-ZFN and right-ZFN vectors were linearized by XhoI and transcribed *in vitro* with T3-RNA-polymerase (Promega) in the presence of m7G(5′)ppp(5′)G to synthesize capped RNA transcripts. The RNA transcripts were precipitated with absolute ethanol, washed and resuspended in RNase-free water (GIBCO). The mRNA concentration was adjusted to 20 µg/ml or 200 µg/ml and the left- and right-ZFN mRNAs were mixed as 1∶1. The RNA solutions were stored at −80°C until use.

### Microinjection of mRNA into Zygote

Following the guidelines for animal experiments at The University of Tokyo, sexually immature female C57BL/6NCr mice (4–5 weeks olds) were superovulated by intraperitoneal injection of 7.5 IU eCG followed by 7.5 IU hCG at an interval of 48 h, and mated overnight with C57BL/6NCr male mice that were more than 10 weeks old. Zygotes were collected after 20 h of hCG injection by oviductal flashing, and pronuclei-formed zygotes were put into the M2 medium. Microinjection was performed using microinjector (Narishige) equipped with microscope. Approximately 4 pl of RNA solution were injected into the cytoplasm of each zygote by continuous pneumatic pressure. After injection, all zygotes were cultured in M16 medium for 24 h and subjected for following experiments.

### Genomic PCR of Single Embryo

For genome DNA collection, an individual 2-cell embryo was put in 10 µl of Ex Taq buffer (RR001B, TaKaRa), digested with 1 µg/µl of Proteinase K at 60°C for 30 min and heat-inactivated at 95°C for 10 min. The embryo lysate solutions were subjected to PCR on the condition of Table 4 using the primer sets in Table 5. The PCR products were purified by agarose gel electrophoresis, then extracted and sequenced as described above.

**Table 4:** Genomic PCR conditions.

PCR mixture	Reaction conditions	
2 µl of 10X Ex Taq Buffer	95°C, 5 min	
2.4 µl of dNTP Mixture (2.5 mM each)	94°C, 30 sec	X 40 cycles for 2-cell embryo or X 35 cycles for tale DNA
1.5 µl of 10 µM Forward primer	57°C, 30 sec	
1.5 µl of 10 µM Reverse primer	72°C, 40 sec	
10 µl of embryo lysate or 1 µl of tale DNA solution	72°C, 5 min	
0.6 µl of 5 U/ml Ex Taq		
12 µl of UltraPure Water		

**Table 5:** Primer sets for genomic PCR.

Target gene	Primor sequence		
*Rosa26*	Forward	5′-	ACGTTTCCGACTTGAGTTGC
	Reverse	5′-	ATACTCCGAGGCGGATCAC
			
*Gli3*	Forward	5′-	GTTACTTAAGGGACGTGAAAACTCA
	Reverse	5′-	ACTAAAGTCTGCCCACCCTATACAC
			
*Il2rg*	Forward	5′-	ATGACTAAAACGAAGTGTGCAGAG
	Reverse	5′-	TAGAGAAGGGTTACAAGGCAAAAG
			
*Cdkn1b*	Forward	5′-	TCCAGTACACTTGATCACTGAAG
	Reverse	5′-	CCTGTAGTAGAACTCGGGCAAG

### Immunoblotting

The micro-western blotting was used for the immunoblotting of the zygotes as described in a previous report [35]. Forty zygotes injected with 200 µg/ml mRNA solutions were used in each lane. The antibodies used were anti-Flag M2 monoclonal antibody (F1804, Sigma-Aldrich) and anti-α tubulin monoclonal antibody (T5168, Sigma-Aldrich). To visualize the protein-bound antibodies, horseradish peroxidase (HRP)-conjugated anti-mouse IgG (Jackson ImmunoReserch Laboratories, Inc., West Grove, PA) was used as a second layer, followed by detection procedure using an ECL detection kit (Amersham-Pharmacia) according to the manufacture’s protocol.

### Immunocytochemistry

After 4 h of mRNA injection, zygotes were fixed in 3.7% paraformaldehyde and permeabilized in 0.2% triton X-100. These zygotes were treated with anti-Flag M2 monoclonal antibody (F1804, Sigma-Aldrich) overnight. After washing, the zygotes were incubated in fluorescein-isothiocyanate (FITC)-conjugated anti-mouse IgG (55494, MP Biomedicals) for 60 min. DNA was visualized by propidium iodide staining, and then examined under a confocal laser scanning microscope (LSM510-V2.01, Axioplan MOT; Carl Zeiss, Oberkochen, Germany).

### T7 Endonuclease I Assay

Genome DNAs were obtained from ten 2-cell embryos injected with 20 µg/ml mRNA solutions as described above, and subjected to PCR using the primer sets shown in Table 3. The purified PCR products were incubated at 95°C for 10 min, then cooled to 85°C at −2°C/sec and to 25°C at −0.5°C/sec for annealing of intact and mutated DNA strands. The re-annealed products were incubated with 2 U of T7 endonuclease I at 37°C for 3 h, then subjected to agarose gel electrophoresis.

### Embryo Transfer and Genotyping of Pups

Two-cell embryos injected with 20 µg/ml ZFN mRNA solutions were transferred into the oviductal ampullas (10–17 embryos per oviduct) of 8-week-old female ICR mouse mated the previous night by vasectomized ICR males. After birth, approximately 1 mm of tail tips were obtained from the 4-day-old pups. Genome DNA was extracted from the tail tips and subjected to PCR on the condition of Table 4 using the primers shown in Table 5. PCR products were purified by agarose gel electrophoresis, and the extracted fragments were directly sequenced as described above.

## Results

### Construction of ZFNs by OLTA

**Figure 2:**
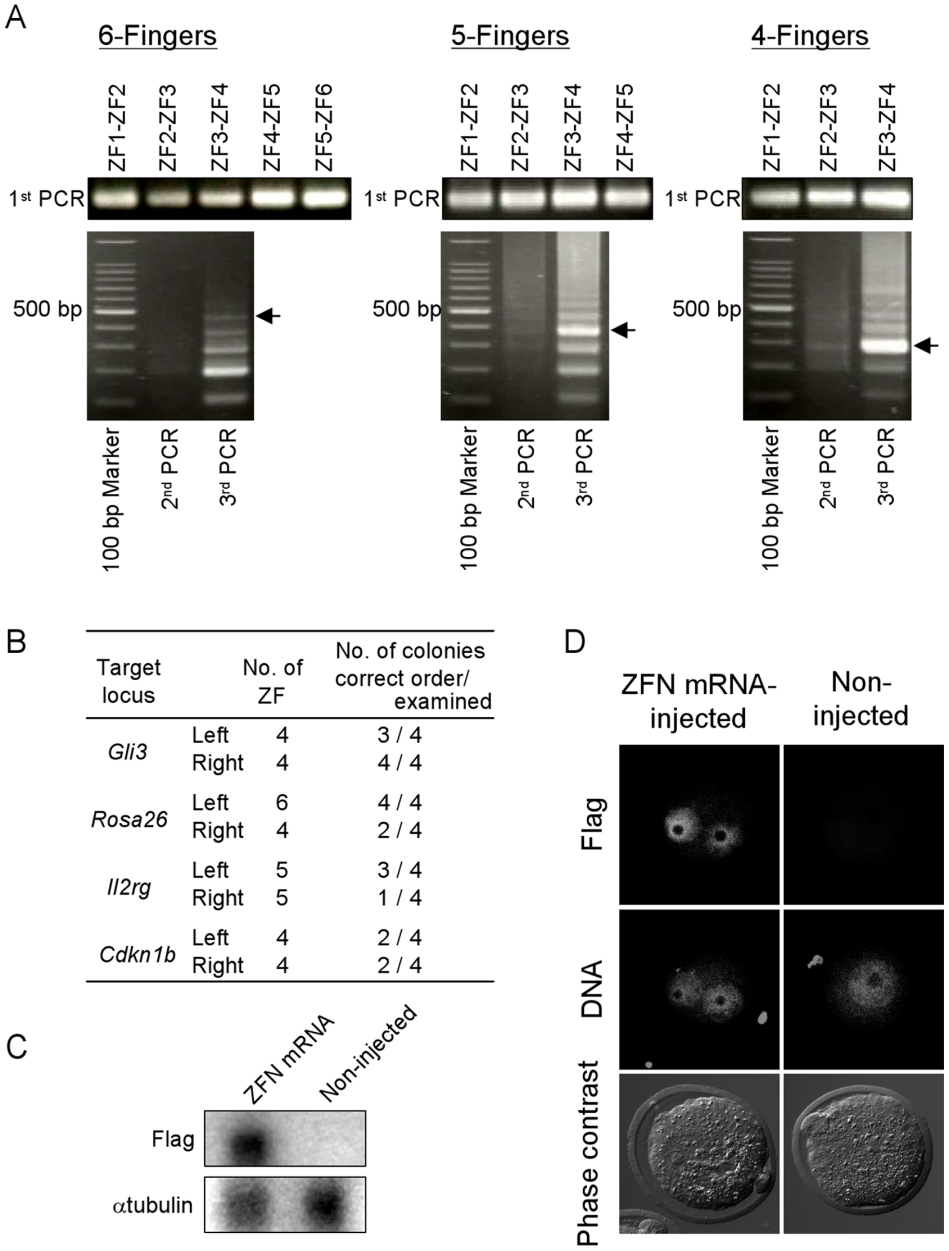
The construction and expression of ZFNs. (A) Three-step PCR for the construction of DNA-binding domain of ZFs. PCR products for each PCR step for 6-finger ZF (left-ZF of *Rosa26*), 5-finger ZF (left-ZF of *Il2rg*), and 4-finger ZF (left-ZF of *Gli3*) are shown. Arrows indicate the intended base pairs of PCR products. (B) Construction efficiencies of left- and right-ZFNs for 4 target loci. (C, D) The expression of constructed ZFN in mouse zygotes. ZFN mRNA at 200 µg/ml (100 µg/ml each for right- and left-ZFNs) were injected into the cytoplasm of mouse zygotes and after 4 h, ZFN protein expression was confirmed by immunoblotting (C) and immunocytochemistry (D) with anti-Flag antibody. Alpha-tubulin immunoblotting is shown as the internal control, and DNA was stained by propidium iodide.

At first, we examined whether the intended ZF could be produced efficiently by overlap PCR utilizing DNA-recognition helices as overlap regions. Short PCR primers consisting of a 21-bp DNA-recognition helix reported previously [16] and an 8-bp ZF common region were designed (Table 2) and partial ZF fragments extending from the DNA-recognition helix to the next DNA-recognition helix, were synthesized by 1st PCR using ZF-template vector and a forward primer (Fw) of one DNA-recognition helix and a reverse primer (Rv) of the next DNA-recognition helix. For the production of *Gli3* left-ZFN, for example, GCC-Fw and TGG-Rv, TGG-Fw and CTG-Rv, and CTG-Fw and GAG-Rv were used as primer sets and 3 partial ZF fragments of ZF1-ZF2, ZF2-ZF3 and ZF3-ZF4 were synthesized. As shown in Figure 2A 1st PCR, all partial ZF fragments were successfully synthesized from the PCR primer sets shown in Table 2. The 1st PCR products were purified by agarose gel electrophoresis and extracted, and then the 3–5 partial ZF fragments (equivalent to 4–6 ZFs) were subjected to a 2nd PCR without a PCR primer (overlap extension PCR) in order to elongate the PCR products. A 3rd PCR was performed using diluted whole 2nd PCR products without purification and extraction, and PCR primers for both ends, for example GCC-Fw and GAG-Rv for *Gli3* left-ZFN. Although no obvious band was observed from the 2nd PCR products (Figure 2A, 2nd PCR), the electrophoresis of the 3rd PCR products showed ladder bands including one at the intended molecular weight (Figure 2A, 3rd PCR). The putative intended molecules extracted from the correct bands of the gels were ligated with platform vectors by TA-cloning; then the ligated vectors were mixed with competent cells and plated. These processes were competed within one day as shown in Table 6. On the following day, colony PCR was performed using T3-promoter primer as forward primer and the reverse primer of 3rd PCR, and each four colonies showing the correct molecular weight and correct direction were selected from each plate. As shown in Figuer 2B, 1 to 4 of the 4 colonies had the intended ZF sequences after sequencing without any reference to the number of ZFs in each array. These results show that the vectors of ZFNs composed of 4–6 ZFs can be produced efficiently by the combination of overlap PCR, utilizing DNA-recognition helices as overlap regions, and TA-cloning. *In vitro* transcribed mRNAs of each ZFN set were injected into mouse zygotes, and the protein expressions of ZFN were observed by western blotting at the correct molecular weight (Figure 2C) and by immunocytochemistry in the pronuclei at 4 h after microinjection (Figure 2D), indicating that the ZFN protein could stably exist in the nucleus in the zygote stage.

**Table 6:** Time schedule for OLTA.

Procedure	Time required (h)
Day 1	
1st PCR	1
Gel purification	1
2nd PCR	] 1
3rd PCR	
Gel purification	1.5
Ligation	0.5–1
Transformation, Plating	0.5
Day 2	
Colony PCR	2
Gel electrophorasis	1
Culture of candidate colonies	O/N*
Day 3	
Plasmid DNA isolation	1
Sequencing reaction	6

* Overnight.

### Functional Analysis of the Constructed ZFNs

**Figure 3:**
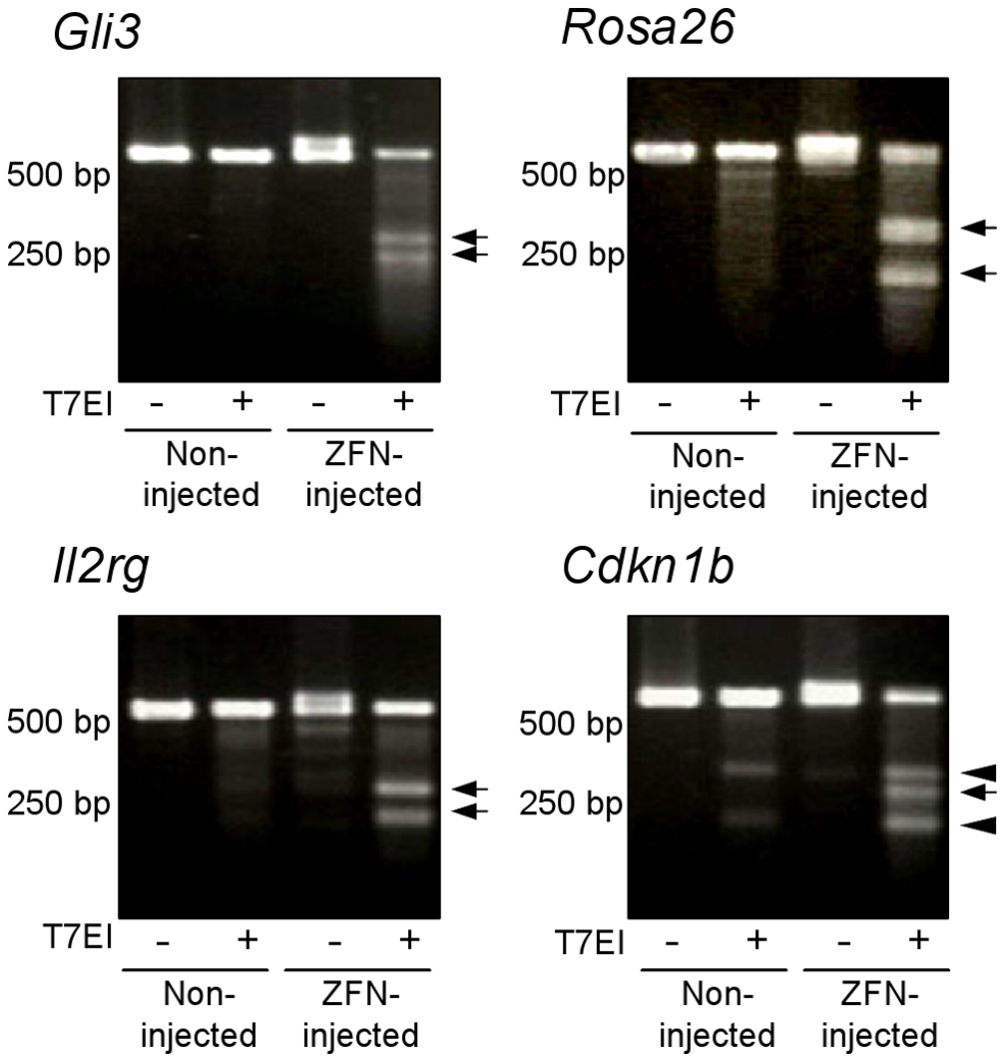
Functional analysis of the constructed ZFNs in preimplantation mouse embryos. Each of ten 2-cell embryos injected with 20 µg/ml of ZFN mRNAs (10 µg/ml each for right- and left-ZFNs) were subjected to T7 endonuclease I assay. Arrows indicate the mismatch-digestion fragments derived from ZFN-induced genome mutation at the target loci. The other fragments digested at the provable SNP sites are indicated by arrowheads.

In order to evaluate the site-directed nuclease activity of the constructed ZFNs, ZFN mRNA sets against four different genome loci, *Rosa26*, *Gli3*, *Il2rg* and *Cdkn1b*, were injected into zygotes and the induction of mutations on the target loci were observed after 24 h of injection. First, PCR for the target loci was performed using 10 embryos, and the PCR products were denatured, re-annealed and treated with T7 endonuclease I, which digests the mismatched base pair. As a result, short fragments caused by mismatch digestion were observed in the ZFN mRNA-injected groups of all target loci (Figure 3), suggesting the digestion of the target loci by the constructed ZFNs. Then, these PCR products were directly sequenced using each forward primer. Microinjection of 200 µg/ml ZFN mRNA against *Gli3* and *Rosa26* resulted in target-site mutations in higher efficiency than microinjection of 20 µg/ml ZFN mRNA (Table 7), however even in the case of 20 µg/ml ZFN mRNA injection, mutated embryos were present at target loci for all ZFNs (Table 7). These results indicate that all of the constructed ZFNs could function as site-directed endonucleases.

**Table 7:** ZFN-induced site-directed mutations in mouse 2-cell embryos.

Target gene	ZFN concentration (µg/ml)	No. of embryos		Sequence of target site*	Modification (S, D, I)**	Number of embryos
		Examined	Mutated			
*Gli3*	200	16	3	AGGCCCACAGCTCTACGGCGACTGAGAGGAAG	WT	13
				AGCCCCACAGCTCTtCGGCGACTGAGAGGAAG	1 S	1
				AGGCCCCCAGCTCT–GCGACTGAGAGGAAG	3 D	1
				AG––––––––––	142 D	1
	20	32	3	AGGCCCACAGCTCTACGGCGACTGAGAGGAAG	WT	29
				AGGCCCACAGCTCT-CGGCGACTGAGAGGAAG	1 D	1
				AGG–––––-GACTGAGAGGAAAG	16D	1
				AGGCCCACAGtTtT–GCGACTGAGAGGAAG	3 D, 2 S	1
*Rosa26*	200	9	9	CTGCAACTCCAGTCTTTCTAGAAGATGGGCGGGAGTCT	WT	0
				CTGCAACTCCAGTCTTTCTAGA–TGGGCGGGAGTCT	3 D	4
				CTGCAACTCCAG––––ATGGtCGGGAGTCT	12 D	1
				CTGCAACTCCAGGCTTTCTA–––––-CT	16 D	1
				CTGCAACTCCAG––––––––TCT	23 D	1
				CTGCAAC––––––––––-	122 D	1
				–––––––––––AGTCT	146 D	1
	20	31	5	CTGCAACTCCAGTCTTTCTAGAAGATGGGCGGGAGTCT	WT	26
				CTGCAACTCCAGTCTTTCTAGAA–TGGGCGGGAGTCT	2 D	1
				CTGCAACTCCAGTCTTTCTAGAA–GGGCGGGAGTCT	3 D	1
				CTGCAACTCCAGTCTTTCTAGA–TGGGCGGGAGTCT	3 D	2
				CTGCAACTCCAGTCTTT––-ATGGGCGGGAGTCT	7 D	1
*Il2rg*	20	30	4	ACCAACCTCACGCTGCACTATAGGTATGAGAAGGGGGA	WT	26
				ACCAACCTCACGCTGCAC–AGGTATGAGAAGGGGGA	3 D	1
				ACCAACCTCACGCTGCAC–-GGTATGAGAAGGGGGA	4 D	1
				ACCAACCTCACGCTGCA–––TGAGAAGGGGGA	9 D	1
				ACCCACCTCACGCTCCAtcacaaccttttgcctGGGGA	16 I	1
*Cdkn1b*	20	39	3	GGTCCACACCCGCCCGAGGAGGAAGATGTCA	WT	36
				GGTCCACACCCGCCC-AGGAGGAAGATGTCA	1 D	1
				GGTCCACACCCGCC–AGGAGGAAGATGTCA	2 D	1
				GGTCCACACCCaCCgGAGGAGGAAGATGTCA	2 S	1

* Under lines indicate the target sites of ZFNs. Small letters and hyphen show the different and deleted nucleotides compared to the WT sequences, respectively.

** S: substitution, D: deletion, I: insertion.

### Generation of Site-directed Mutated mice Using the Constructed ZFN

Finally, we examined the toxicity of constructed ZFNs and whether the constructed ZFN vectors were useful for the generation of site-directed mutated mice. About 80% of the embryos injected with water or 20 µg/ml of *Gli3*, *Rosa26* or *Il2rg* ZFN mRNA developed to become blastocysts (Table8). However, the embryos injected with *Cdkn1b* ZFN mRNA developed normally up to the 2-cell stage, but many of them stopped thereafter (Table 8). Agreeing with these results, when the embryos injected with 20 µg/ml of ZFN mRNA were transferred into the oviducts of recipient mice at the 2-cell stage, site-directed mutated mice were obtained in every case other than *Cdkn1b* ZFN-mRNA injection (Table 9), These results indicate that although *Cdkn1b* ZFN had some toxicities for embryo development, most of the ZFN vectors constructed by OLTA can be used for generation of site-directed mutated mice.

**Table 8:** *In vitro* development of ZFN-injected mouse embryos.

Target gene	No. (%) of embryos				
	total	2-cell		Blastocyst	
Non-injected	27	27	(100)	27	(100)
Water-injected	40	38	(95)	32	(80)
*Gli3*	165	148	(89.7)	125	(75.8)
*Rosa26*	68	63	(92.6)	59	(86.8)
*Il2rg*	83	72	(86.7)	64	(77.1)
*Cdkn1b*	48	40	(83.3)	11	(22.9)

**Table 9:** ZFN-induced site-directed mutations in new-born mouse.

Target gene	2-cell embryos transferred	Born	Mutated	Sequence of terget site*	Modification(D, I)**	Number of embryos
*Gli3*	95	22	5	AGGCCCACAGCTCTACGGCGACTGAGAGGAAGAAAGC	WT	17
				AGGCCCACAGCTCT-CGGCGACTGAGAGGAAGAAAGC	1 D	1
				AGGCCCACAGCTCTA-GGCGACTGAGAGGAAGAAAGC	1 D	1
				AGGCCCACAGCTCTA–GCGACTGAGAGGAAGAAAGC	2 D	1
				AGGCCCACAGCTCT––GACTGAGAGGAAGAAAGC	5 D	1
				AGGCCCACAGCTCTACGGggCGACTGAGAGGAAGAAAGC	2 I	1
*Rosa26*	42	7	1	CTGCAACTCCAGTCTTTCTAGAAGATGGGCGGGAGTCT	WT	6
				CTGCAACTCCAGTCTTTCTA––TGGGCGGGAGTCT	5 D	1
*Il2rg*	40	8	1	ACCAACCTCACGCTGCACTATAGGTATGAGAAGGGGGA	WT	7
				ACCAACCTCACGCTG––-GGTATGAGAAGGGGGA	7 D	1
*Cdkn1b*	61	0	0	GGTCCACACCCGCCCGAGGAGGAAGATGTCA	WT	-

* Under lines indicate the target sites of ZFNs. Small letters and hyphen show the inserted and deleted nucleotids compared to the WT seuences, respectively.

** D: deletion, I: insertion.

## Discussion

Although ZFN is a useful tool for site-directed genome modification, the development of useful construction methods that are easy, inexpensive and repeatedly usable for multiple kinds of ZFN should contribute to the further widespread use of this technology. In this study, we established a novel construction method named “OLTA”, in which the intended DNA-binding domains, composed of 4 to 6 ZFs, were synthesized by overlap extension PCR of partial ZF fragments and joined with a nuclease vector by TA cloning. Using this method, we succeeded in constructing beneficial ZFN vectors in a low-cost manner in a short period of time. All ZFNs constructed by OLTA in the present study functioned as site-directed nucleases, and a genetically modified mouse was successfully generated using the constructed ZFN.

The most common construction method for ZFN thus far has been the assembly of ZF modules from a prepared ZF vector library [24], [25] or the overlapping of synthetic long oligonucleotides by PCR [16], [26]. In the reported overlap-PCR method for ZFN construction, a DNA-binding domain of ZFN was divided into several synthetic long (60 bp<) oligonucleotide fragments having overlap sequences in both ends, and these fragments were combined by PCR utilizing the overlap sequences. In this method, each DNA-recognition helix was coded at various positions in each fragment. In order to combine the multiple fragments into a correct order by PCR at once, the overlap sequences of each fragment were selected from different sites of ZF domain. Therefore an oligonucleotide coding one DNA-recognition helix could be used for only a specific position, and as a result, new long oligonucleotides were required each time for changing the ZF position. In contrast, OLTA amplifies common ZF framework by PCR using short (30 bp>) primers consisting of a 21-bp DNA-recognition helix and an 8-bp ZF common region, and these partial ZF fragments extending from a DNA-recognition helix to the next DNA-recognition helix are combined by PCR utilizing the overlapped DNA-recognition helix sequences. Therefore, once prepared, the primers corresponding to each DNA triplet can be used repeatedly for the construction of other ZFN vectors without position limitation, in every case of the present study, partial ZF fragments were successfully synthesized precisely by the 1st PCR using each set of primers shown in Table 2 (Figure 2A). This result indicates that partial ZF fragments including various types of DNA recognition helices can be synthesized by the present PCR-primer conditions; the number of nucleotide differences with the template vector are 11 or less and 8 bp of 3′-end are completely complementary. Although only 21 kinds of DNA-recognition helices were synthesized in this study, more than 46 other kinds of DNA-recognition helices specific for various DNA triplets have been reported to date [16], [17]. All of these helices can be expected to be synthesized by OLTA, because their primer sets satisfy the above-mentioned PCR-primer conditions. Therefore, OLTA should be considered as a versatile and powerful method for ZFN construction.

It is highly conceivable that the numerical difference in ZFs have affected the ZFN recruiting efficiency for the correct position and mutation rates of endogenous target genome loci. The method of ZF-module assembly has the merit to combine ZFNs without number limitation at least in principle. However, this method requires consecutive restriction and ligation reactions, which make this method complex and time consuming. In contrast, ZFN vectors can be synthesized within a single day by OLTA; the construction process is completed within three days even if the transformation and the sequencing of ZFN vectors are included as shown in Table 6. Furthermore, the fact that one month was sufficient for the generation of mice with site-directed mutations, from the construction of the ZFN to the obtaining of pups, indicates that an extremely short-term generation of genome-modified animals is possible with OLTA. With regard to the overlapping of long oligonucleotides, ZFNs can be synthesized in a short term as with OLTA [26], but the reported numbers of ZFs in DNA-binding domains have been confined to 3. The past routine for the preparation of a 6-finger protein, for example, was to make 3-finger proteins by overlap PCR and then to ligate the two 3-finger proteins together into a 6-finger protein. In the case of OLTA, at least 5 partial ZF fragments were successfully joined by 2nd and 3rd PCRs. The vector construction efficiencies, 1/4 to 4/4 (25–100%) in OLTA (Figure 2B), were almost the same as those (17–75%) reported previously. Thus, the OLTA method compensates adequately for the weak points of traditional construction methods.

In the present study, all four ZFN sets constructed by OLTA functioned as site-directed nucleases for genome DNA in mouse 2-cell embryos. The most likely explanation for the high ZFN activity on the endogenous target loci in the present study might be the presence of 4 to 6 ZFs in the present ZFN sets instead of 3 ZFs in the previous method. Previous reports studying the effects of ZF numbers on the target recognition efficiencies have shown enhancement of recognition efficiency by more than 4 ZFs [28], [36], [37]. Further, there are several reports about the direct-mutagenesis of mouse embryonic *Rosa26* locus using ZFN that have different numbers of fingers. Meyer et al generated site-directed mutated mice using ZFN sets that have 4- and 6-fingers for the target sequence the same as us, resulted that 22% of pups showed NHEJ-mediated mutation [4]. On the other hand, Hermann et al. reported that several 3-fingers of ZFN sets designed by OPEN generated only 0 to 7.4% of mutated pups [5]. In the present study, 4- and 6-finger ZFN sets against the *Rosa26* locus generated 14.3% of mutated pup (Table 9), which efficiency is higher as well as Meyer’s report than Hermann’s. These results may support the hypothesis that the numbers of fingers increase the efficiencies of the mutation induction. Another reason might be the use of relatively high ZFN concentration for the evaluation of ZFN activities. It is well known that ZFNs have off-target effects, non-specific digestions of non-target sites, and this has become a general problem for ZFN experiments [38]. A previous report showed that off-target incidences increase depending on the concentration of ZFN [39]. The culture cells attacked by the off-target effects should be removed from the culture system by the induction of apoptosis even if their target loci were digested correctly. Therefore, the concentration of ZFNs was usually kept as low as possible to exert only the desired effect. On the other hand, we used mouse fertilized embryos that can develop to the 2-cell stage by the help of maternal factors even in the presence of off-target effects so as to evaluate the ZFN activity free of influence from off-target effects. In fact, although ZFNs injected at a concentration of 200 µg/ml showed higher mutation efficiency than those at 20 µg/ml, development stopped at the 2-cell stage and blastocysts were not observed. This failure of oocytes do develop further is most likely due to the off-target effect, by excessive ZFN expression.

The embryos injected with 20 µg/ml, all with the exception of *Cdkn1b*, ZFN mRNA successfully developed to pup that had mutations at the correct target loci. This result suggests that off-target effects can be evaded by using a 4 or more ZF-containing ZFN mRNA set at a concentration of 20 µg/ml. For the evasion of off-target toxicity of ZFN, another effective solution is thought to increase the number of ZFs in each array and elevating the ZFN specificity. Comparison study using human cells revealed that 3-finger-ZFNs showed off-target cutting at 31 loci whereas 4-finger-ZFNs showed 9 loci [39]. Until now, only 3 to 6 ZF-containing ZFNs have been used for site-directed genome modification, and the efficiency of more than 6 ZF-containing ZFNs has never been reported. One reason for this may have been the difficulty of constructing a long ZFN using conventional methods. In contrast, in the present electrophoretic patterns of the 3rd PCR, bands of longer than 6 ZF were observed (Figure 2A), suggesting that the OLTA method can be adopted for the construction of ZFNs consisting of more than 6 ZFs–although some DNA-binding domains obtained by OLTA in the present study showed incorrect ZF order. It is necessary to examine how many ZFs can be connected precisely by OLTA and confirm the correlation of ZF number to binding specificity and efficiency, in especially those greater than six.

In conclusion, the present study indicates that OLTA can be applied as a new ZFN construction method that is easy, non-expensive and available for repeated use for multiple kinds of ZFNs, thereby compensating for the weak points of the conventional methods. Recently, efficient construction methods for TAL-effecter nuclease, which is another artificial nuclease, have been reported [3]. Comparison of various kinds of ZFNs and TALENs constructed by various methods including OLTA is expected to contribute to the advancement of artificial nuclease technologies and genome-editing fields.

## Supporting Information

Figure S1 **DNA and protein sequences of KK or EL mutation-induced Platform vector. Boxes showed the T3 promoter site and underline showed the XhoI site for linearization.**
(DOC)

Figure S2 **DNA sequence of a partial ZF sequence of template vector. Boxes showed the DNA recognition helices.**
(DOC)

## References

[1] Durai S, Mani M, Kandavelou K, Wu J, Porteus MH, et al (2005) Zinc finger nucleases: custom-designed molecular scissors for genome engineering of plant and mammalian cells. Nucleic Acids Res 33: 5978–5990. 16251401 10.1093/nar/gki912 PMC1270952

[2] Porteus MH, Carroll D (2005) Gene targeting using zinc finger nucleases. Nat Biotechnol 23: 967–973. 16082368 10.1038/nbt1125

[3] Perez-Pinera P, Ousterout DG, Gersbach CA (2012) Advances in targeted genome editing. Curr Opin Chem Biol 16: 268–277. 22819644 10.1016/j.cbpa.2012.06.007 PMC3424393

[4] Meyer M, de Angelis MH, Wurst W, Kühn R (2010) Gene targeting by homologous recombination in mouse zygotes mediated by zinc-finger nucleases. Proc Natl Acad Sci U S A 107: 15022–15026. 20686113 10.1073/pnas.1009424107 PMC2930558

[5] Hermann M, Maeder ML, Rector K, Ruiz J, Becher B, et al (2012) Evaluation of OPEN zinc finger nucleases for direct gene targeting of the ROSA26 locus in mouse embryos. PLoS One 7: e41796. 22970113 10.1371/journal.pone.0041796 PMC3435328

[6] Geurts AM, Cost GJ, Freyvert Y, Zeitler B, Miller JC, et al (2009) Knockout rats via embryo microinjection of zinc-finger nucleases. Science 325: 433. 19628861 10.1126/science.1172447 PMC2831805

[7] Hauschild J, Petersen B, Santiago Y, Queisser AL, Carnwath JW, et al (2011) Efficient generation of a biallelic knockout in pigs using zinc-finger nucleases. Proc Natl Acad Sci U S A 108: 12013–12017. 21730124 10.1073/pnas.1106422108 PMC3141985

[8] Young JJ, Cherone JM, Doyon Y, Ankoudinova I, Faraji FM, et al (2011) Efficient targeted gene disruption in the soma and germ line of the frog Xenopus tropicalis using engineered zinc-finger nucleases. Proc Natl Acad Sci U S A 108: 7052–7057. 21471457 10.1073/pnas.1102030108 PMC3084115

[9] Yu S, Luo J, Song Z, Ding F, Dai Y, et al (2011) Highly efficient modification of beta-lactoglobulin (BLG) gene via zinc-finger nucleases in cattle. Cell Res 21: 1638–1640. 21912434 10.1038/cr.2011.153 PMC3364726

[10] Meng X, Noyes MB, Zhu LJ, Lawson ND, Wolfe SA (2008) Targeted gene inactivation in zebrafish using engineered zinc-finger nucleases. Nat Biotechnol 26: 695–701. 18500337 10.1038/nbt1398 PMC2502069

[11] Cai CQ, Doyon Y, Ainley WM, Miller JC, Dekelver RC, et al (2008) Targeted transgene integration in plant cells using designed zinc finger nucleases. Plant Mol Biol 69: 699–709. 19112554 10.1007/s11103-008-9449-7

[12] Wood AJ, Lo TW, Zeitler B, Pickle CS, Ralston EJ, et al (2011) Targeted genome editing across species using ZFNs and TALENs. Science 333: 307. 21700836 10.1126/science.1207773 PMC3489282

[13] Bibikova M, Beumer K, Trautman J, Carroll D (2003) Enhancing Gene Targeting with Designed Zinc Finger Nucleases. Science 300: 764. 12730594 10.1126/science.1079512

[14] Maeder ML, Thibodeau-Beganny S, Osiak A, Wright DA, Anthony RM, et al (2008) Rapid “open-source” engineering of customized zinc-finger nucleases for highly efficient gene modification. Mol Cell 31: 294–301. 18657511 10.1016/j.molcel.2008.06.016 PMC2535758

[15] Miller JC, Holmes MC, Wang J, Guschin DY, Lee YL, et al (2007) An improved zinc-finger nuclease architecture for highly specific genome editing. Nat Biotechnol 25: 778–785. 17603475 10.1038/nbt1319

[16] Carroll D, Morton JJ, Beumer KJ, Segal DJ (2006) Design, construction and *in vitro* testing of zinc finger nucleases. Nat Protoc 1: 1329–1341. 17406419 10.1038/nprot.2006.231

[17] Mackay JP, Segal DJ (2010) Engineered Zinc Finger Proteins. Humana Press: New York.

[18] Sander JD, Dahlborg EJ, Goodwin MJ, Cade L, Zhang F, et al (2011) Selection-free zinc-finger-nuclease engineering by context-dependent assembly (CoDA). Nat Methods 8: 67–69. 21151135 10.1038/nmeth.1542 PMC3018472

[19] Gupta A, Christensen RG, Rayla AL, Lakshmanan A, Stormo GD, et al (2012) An optimized two-finger archive for ZFN-mediated gene targeting. Nat Methods 9: 588–590. 22543349 10.1038/nmeth.1994 PMC3443678

[20] Mandell JG, Barbas CF 3rd (2006) Zinc Finger Tools: custom DNA-binding domains for transcription factors and nucleases. Nucleic Acids Res 34: W516–523. 16845061 10.1093/nar/gkl209 PMC1538883

[21] Fu F, Sander JD, Maeder M, Thibodeau-Beganny S, Joung JK, et al (2009) Zinc Finger Database (ZiFDB): a repository for information on C2H2 zinc fingers and engineered zinc-finger arrays. Nucleic Acids Res 37: D279–283. 18812396 10.1093/nar/gkn606 PMC2686427

[22] Sander JD, Maeder ML, Reyon D, Voytas DF, Joung JK, et al (2010) ZiFiT (Zinc Finger Targeter): an updated zinc finger engineering tool. Nucleic Acids Res 38: W462–268. 20435679 10.1093/nar/gkq319 PMC2896148

[23] Cradick TJ, Ambrosini G, Iseli C, Bucher P, McCaffrey AP (2011) ZFN-site searches genomes for zinc finger nuclease target sites and off-target sites. BMC Bioinformatics 12: 152. 21569489 10.1186/1471-2105-12-152 PMC3113941

[24] Wright DA, Thibodeau-Beganny S, Sander JD, Winfrey RJ, Hirsh AS, et al (2006) Standardized reagents and protocols for engineering zinc finger nucleases by modular assembly. Nat Protoc 1: 1637–1652. 17406455 10.1038/nprot.2006.259

[25] Gonzalez B, Schwimmer LJ, Fuller RP, Ye Y, Asawapornmongkol L, Barbas CF 3rd (2010) Modular system for the construction of zinc-finger libraries and proteins. Nat Protoc 5: 791–810. 20360772 10.1038/nprot.2010.34 PMC2855653

[26] Osborn MJ, DeFeo AP, Blazar BR, Tolar J (2011) Synthetic zinc finger nuclease design and rapid assembly. Hum Gene Ther 22: 1155–1165. 21663559 10.1089/hum.2011.072 PMC4076977

[27] Defrancesco L (2011) Move over ZFNs. Nat Biotechnol 29: 681–684. 21822235 10.1038/nbt.1935

[28] Perez-Pinera P, Ousterout DG, Brown MT, Gersbach CA (2012) Gene targeting to the *ROSA26* locus directed by engineered zinc finger nucleases. Nucleic Acids Res 240: 3741–3752. 10.1093/nar/gkr1214 PMC3333879 22169954

[29] Mashimo T, Takizawa A, Voigt B, Yoshimi K, Hiai H, et al (2010) Generation of knockout rats with X-linked severe combined immunodeficiency (X-SCID) using zinc-finger nucleases. PLoS One 5: e8870. 20111598 10.1371/journal.pone.0008870 PMC2810328

[30] Guo J, Gaj T, Barbas CF 3rd (2010) Directed evolution of an enhanced and highly efficient FokI cleavage domain for zinc finger nucleases. J Mol Biol 400: 96–107. 20447404 10.1016/j.jmb.2010.04.060 PMC2885538

[31] Sallés FJ, Strickland S (1999) Analysis of poly(A) tail lengths by PCR: the PAT assay. Methods Mol Biol 118: 441–448. 10549542 10.1385/1-59259-676-2:441

[32] Elrod-Erickson M, Rould MA, Nekludova L, Pabo CO (1996) Zif268 protein-DNA complex refined at 1.6 A: a model system for understanding zinc finger-DNA interactions. Structure 4: 1171–1180. 8939742 10.1016/s0969-2126(96)00125-6

[33] Wolfe SA, Greisman HA, Ramm EI, Pabo CO (1999) Analysis of zinc fingers optimized via phage display: evaluating the utility of a recognition code. J Mol Biol 285: 1917–1934. 9925775 10.1006/jmbi.1998.2421

[34] Wolfe SA, Grant RA, Elrod-Erickson M, Pabo CO (2001) Beyond the “recognition code”: structures of two Cys2His2 zinc finger/TATA box complexes. Structure 9: 717–723. 11587646 10.1016/s0969-2126(01)00632-3

[35] Naito K, Kagii H, Iwamori N, Sugiura K, Yamanouchi K, et al (1999) Establishment of a small-scale Western blotting system named as “micro-Western blotting” for mammalian ova analysis. J Mamm Ova Res 16: 154–157.

[36] Shimizu Y, Şöllü C, Meckler JF, Adriaenssens A, Zykovich A, et al (2011) Adding fingers to an engineered zinc finger nuclease can reduce activity. Biochemistry 50: 5033–5041. 21528840 10.1021/bi200393g PMC3110833

[37] Bhakta MS, Henry IM, Ousterout DG, Das KT, Lockwood SH, et al.. (2013) Highly active zinc-finger nucleases by extended modular assembly. Genome Res In press. 10.1101/gr.143693.112 PMC3589541 23222846

[38] Radecke S, Radecke F, Cathomen T, Schwarz K (2010) Zinc-finger nuclease-induced gene repair with oligodeoxynucleotides: wanted and unwanted target locus modifications. Mol Ther 18: 743–753. 20068556 10.1038/mt.2009.304 PMC2862519

[39] Pattanayak V, Ramirez CL, Joung JK, Liu DR (2011) Revealing off-target cleavage specificities of zinc-finger nucleases by *in vitro* selection. Nat Methods 8: 765–770. 21822273 10.1038/nmeth.1670 PMC3164905

