# Supplementary material for: Repeatable Construction Method for Engineered Zinc Finger Nuclease Based on Overlap Extension PCR and TA-Cloning
Source: PLoS One. 2013 Mar 25;8(3):e59801. doi: 10.1371/journal.pone.0059801 (PMC3607563; doi:10.1371/journal.pone.0059801)
Supplement: Figure S1 — DNA and protein sequences of KK or EL mutation-induced Platform vector. Boxes showed the T3 promoter site and underline showed the XhoI site for linearization. (DOC) [file pone.0059801.s001.doc]

**Figure S1**

***KK-Platform-vector***

AATTAACCCTCACTAAAGGGAACAAAAGCTGGAGCTCCACCGCGGTGGCGGCCGCTCTAGCCCGGGCGGATCCACC

ATGGATTACAAGGATGACGACGATAAGATCTCCCCCAAGAAGAAGAGGAAGGTGGGATCACAGAAGCCC

M D Y K D D D D K I S P K K K R K V G S Q K P

TTCCAGTGCCGCATCTGCATGCGCAACTTCAGCTGCCCGTATACCAAGATCCACCTGCGGGGATCCCAA

F Q C R I C M R N F S C P Y T K I H L R G S Q

CTAGTCAAAAGTGAACTGGAGGAGAAGAAATCTGAACTTCGTCATAAATTGAAATATGTGCCTCATGAA

L V K S E L E E K K S E L R H K L K Y V P H E

TATATTGAATTAATTGAAATTGCCAGAAATCCCACTCAGGATAGAATTCTTGAAATGAAGGTAATGGAA

Y I E L I E I A R N P T Q D R I L E M K V M E

TTTTTTATGAAAGTTTATGGATATAGAGGTGAACATTTGGGTGGATCAAGGAAACCGGACGGAGCAATT

F F M K V Y G Y R G E H L G G S R K P D G A I

TATACTGTCGGATCTCCTATTGATTACGGTGTGATCGTGGATACTAAAGCTTATAGCGGAGGTTATAAT

Y T V G S P I D Y G V I V D T K A Y S G G Y N

CTGCCAATTGGCCAAGCAGATGAAATGCAACGATATGTCAAAGAAAATCAAACACGAAACAAACATATC

L P I G Q A D E M Q R Y V K E N Q T R N K H I

AACCCTAATGAATGGTGGAAAGTCTATCCATCTTCTGTAACGGAATTTAAGTTTTTATTTGTGAGTGGT

N P N E W W K V Y P S S V T E F K F L F V S G

CACTTTAAAGGAAACTACAAAGCTCAGCTTACACGATTAAATCATAAGACTAATTGTAATGGAGCTGTT

H F K G N Y K A Q L T R L N H K T N C N G A V

CTTAGTGTAGAAGAGCTTTTAATTGGTGGAGAAATGATTAAAGCCGGCACATTAACCTTAGAGGAAGTG

L S V E E L L I G G E M I K A G T L T L E E V

AGACGGAAATTTAATAACGGCGAGATAAACTTTTGA

R R K F N N G E I N F *

GGTACCTATGGGCACCAAAGAACCTGTAAACGTTATCTTTTTAAATTGAATGTGCACAAATAAAAGTTTGGAAAAGAAAAAAAAAAAAAAAAAAAAAAAAAAAAAAAAAAAAAAAAAAAAAAAAAAAAAAAAAAAAAAAAAAAAAAAAAAAAAAAAAAAAAAAAAAAAAAACTCGAGGTACC

***EL-Platform- vector***

AATTAACCCTCACTAAAGGGAACAAAAGCTGGAGCTCCACCGCGGTGGCGGCCGCTCTAGCCCGGGCGGATCCACC

ATGGATTACAAGGATGACGACGATAAGATCTCCCCCAAGAAGAAGAGGAAGGTGGGATCACAGAAGCCC

M D Y K D D D D K I S P K K K R K V G S Q K P

TTCCAGTGCCGCATCTGCATGCGCAACTTCAGCTGCCCGTATACCAAGATCCACCTGCGGGGATCCCAA

F Q C R I C M R N F S C P Y T K I H L R G S Q

CTAGTCAAAAGTGAACTGGAGGAGAAGAAATCTGAACTTCGTCATAAATTGAAATATGTGCCTCATGAA

L V K S E L E E K K S E L R H K L K Y V P H E

TATATTGAATTAATTGAAATTGCCAGAAATCCCACTCAGGATAGAATTCTTGAAATGAAGGTAATGGAA

Y I E L I E I A R N P T Q D R I L E M K V M E

TTTTTTATGAAAGTTTATGGATATAGAGGTGAACATTTGGGTGGATCAAGGAAACCGGACGGAGCAATT

F F M K V Y G Y R G E H L G G S R K P D G A I

TATACTGTCGGATCTCCTATTGATTACGGTGTGATCGTGGATACTAAAGCTTATAGCGGAGGTTATAAT

Y T V G S P I D Y G V I V D T K A Y S G G Y N

CTGCCAATTGGCCAAGCAGATGAAATGGAACGATATGTCGAAGAAAATCAAACACGAAACAAACATCTC

L P I G Q A D E M E R Y V E E N Q T R N K H L

AACCCTAATGAATGGTGGAAAGTCTATCCATCTTCTGTAACGGAATTTAAGTTTTTATTTGTGAGTGGT

N P N E W W K V Y P S S V T E F K F L F V S G

CACTTTAAAGGAAACTACAAAGCTCAGCTTACACGATTAAATCATATCACTAATTGTAATGGAGCTGTT

H F K G N Y K A Q L T R L N H I T N C N G A V

CTTAGTGTAGAAGAGCTTTTAATTGGTGGAGAAATGATTAAAGCCGGCACATTAACCTTAGAGGAAGTG

L S V E E L L I G G E M I K A G T L T L E E V

AGACGGAAATTTAATAACGGCGAGATAAACTTTTGA

R R K F N N G E I N F *

GGTACCTATGGGCACCAAAGAACCTGTAAACGTTATCTTTTTAAATTGAATGTGCACAAATAAAAGTTTGGAAAAGAAAAAAAAAAAAAAAAAAAAAAAAAAAAAAAAAAAAAAAAAAAAAAAAAAAAAAAAAAAAAAAAAAAAAAAAAAAAAAAAAAAAAAAAAAAAAAACTCGAGGTACC
