# Supplementary material for: Repeatable Construction Method for Engineered Zinc Finger Nuclease Based on Overlap Extension PCR and TA-Cloning
Source: PLoS One. 2013 Mar 25;8(3):e59801. doi: 10.1371/journal.pone.0059801 (PMC3607563; doi:10.1371/journal.pone.0059801)
Supplement: Figure S2 — DNA sequence of a partial ZF sequence of template vector. Boxes showed the DNA recognition helices. (DOC) [file pone.0059801.s002.doc]

**Figure S2**

CGCTCGGATGCGCTTACCCGCCATATCCGCATCCACACAGGCCAGAAGCCCTTCCAGTGTCGAATCTGC

R S D A L T R H I R I H T G Q K P F Q C R I C

ATGCGTAACTTCAGTCGCTCGGATAATCTTGCCCGC

M R N F S R S D N L A R
